# Commentary: Computerised interpretation of fetal heart rate during labour (INFANT): a randomised controlled trial

**DOI:** 10.3389/fphys.2017.00721

**Published:** 2017-09-28

**Authors:** Martin G. Frasch, Geraldine B. Boylan, Hau-tieng Wu, Declan Devane

**Affiliations:** ^1^Department of Obstetrics and Gynecology, University of Washington, Seattle, WA, United States; ^2^Irish Centre for Fetal and Neonatal Translational Research, Cork, Ireland; ^3^Department of Paediatrics and Child Health, University College Cork, Cork, Ireland; ^4^Department of Mathematics and Department of Statistical Science, Duke University, Durham, NC, United States; ^5^School of Nursing and Midwifery, NUI Galway, Galway, Ireland

**Keywords:** labor, fHRV, EEG, acidemia, sampling rate, FHR, neonate

The recent study by the INFANT group reported no evidence of benefit on neonatal outcomes associated with the use of decision-support software in conjunction with cardiotocography (CTG) compared with CTG alone (The INFANT Collaborative Group, 2017). Concerns about the study design have been voiced (Keith, 2017).

Earlier studies comparing continuous CTG with intermittent auscultation during labor have shown CTG during labor to be associated with reduced rates of neonatal seizures, largely of unknown long term consequence, but no clear differences in other measures of neonatal mortality and morbidity. However, continuous CTG is associated with increased operative delivery rates (Alfirevic et al., 2017). We now know that supplementing CTG with decision-support software is unlikely to improve outcomes. Yet, given the absence of any alternative to CTG, for high risk women, it is likely that conventional CTG will retain a firm grip in labor wards.

Efforts should now be directed toward interrogating fetal heart rate variability (fHRV) more deeply and/or measuring and evaluating different physiological parameters of intrapartum fetal well-being. For example, the true predictive ability of fetal ECG can only be determined once it is collected at a sampling rate that preserves more of the underlying physiological information. We have demonstrated this approach to assess the severity of hypoxic ischaemic encephalopathy (HIE) in the immediate newborn period (Goulding et al., 2017). Animal and human studies show that the current mode of ECG acquisition is outdated, imprecise, and discards important predictive information (Durosier et al., 2014; Li et al., 2015). To address this challenge, we recently developed and validated an algorithm for low-cost, portable high quality maternal, and fetal ECG monitoring capable of working with one or two maternal abdominal ECG channels to extract the fetal ECG (Li and Wu, 2017; Wu et al., 2017). While we need at least two channels for the algorithm introduced in (Wu et al., 2017), it could be applied to handle the single channel maternal abdominal ECG signal as generalized in (Li and Wu, 2017). An important challenge is refining the algorithm to perform well in the case of twin pregnancies. Also, for the wide acceptance of the technology, it will have to be considerably less expensive than the currently available fetal ECG monitors.

Surprisingly little research has been done on what is perhaps the most obvious and direct source of predictive information about fetal brain health, the Electroencephalogram (EEG), even though it is very sensitive to hypoxia-ischaemia (Murray et al., 2016; Finn et al., 2017). Using animal models, we have developed and validated a new algorithm for fetal EEG as predictor of academia (Frasch et al., 2015) and are beginning a clinical study using a fetal EEG prototype device (NCT03013569). Our data shows that pathognomonic EEG changes emerge at a pH ≤ 7.20, which the INFANT study reported in at least 12% of all babies.

We expect that direct acquisition of fetal EEG during labor and a more precise acquisition of fetal ECG (and fHRV) offers potential in preventing acidosis and brain injury due to intrapartum hypoxia-ischaemia. Fetal infection is an important contributor to perinatal brain injury and fHRV has been shown to reflect fetal inflammatory response systematically and in an organ-specific manner (Durosier et al., 2015; Frasch et al., 2016; Liu et al., 2016). Prospective studies in large ante- and intrapartum cohorts of pregnant women and fetuses are now needed to validate the utility of fetal ECG and EEG monitors. Such studies will not only yield biomarkers to predict complications during delivery due to underlying infection, hypoxia or acidemia, but also pinpoint the risks for abnormal postnatal developmental trajectories. Knowing on which babies to focus will provide a foundation upon which to build the therapeutic strategies to correct early deviations from healthy developmental trajectories.

## Author contributions

All authors listed have made a substantial, direct and intellectual contribution to the work, and approved it for publication.

### Conflict of interest statement

MF is an inventor of related patent application entitled “EEG Monitor of Fetal Health” including U.S. Patent 9,215,999. MF and HW filed a patent for the aECG method referred to in this commentary. The other authors declare that the research was conducted in the absence of any commercial or financial relationships that could be construed as a potential conflict of interest.
